# Modeling and predicting individual tacit coordination ability

**DOI:** 10.1186/s40708-022-00152-w

**Published:** 2022-02-04

**Authors:** Dor Mizrahi, Ilan Laufer, Inon Zuckerman

**Affiliations:** Department of Industrial Engineering and Management, Ariel University, Ariel, Israel

**Keywords:** Tacit coordination, Decision-making, Cognitive modeling

## Abstract

**Background:**

Previous experiments in tacit coordination games hinted that some people are more successful in achieving coordination than others, although the variability in this ability has not yet been examined before. With that in mind, the overarching aim of our study is to model and describe the variability in human decision-making behavior in the context of tacit coordination games.

**Methods:**

In this study, we conducted a large-scale experiment to collect behavioral data, characterized the distribution of tacit coordination ability, and modeled the decision-making behavior of players. First, we measured the multimodality in the data and described it by using a Gaussian mixture model. Then, using multivariate linear regression and dimensionality reduction (PCA), we have constructed a model linking between individual strategic profiles of players and their coordination ability. Finally, we validated the predictive performance of the model by using external validation.

**Results:**

We demonstrated that coordination ability is best described by a multimodal distribution corresponding to the levels of coordination ability and that there is a significant relationship between the player’s strategic profile and their coordination ability. External validation determined that our predictive model is robust.

**Conclusions:**

The study provides insight into the amount of variability that exists in individual tacit coordination ability as well as in individual strategic profiles and shows that both are quite diverse. Our findings may facilitate the construction of improved algorithms for human–machine interaction in diverse contexts. Additional avenues for future research are discussed.

## Background

A tacit coordination game is one in which two individuals are rewarded for making the same choice from the same set of alternatives, and any form of communication between the players is not allowed or not possible (e.g., [1–4]). Such problems have been formally modeled in game theory as games with multiple Nash equilibria solutions with equal values [5]. Tacit coordination problems can be found in real-life situations such as tacit collusion among retail chains [6], social coordination, allowing, for example, the convergence on a new technological platform [7], and auction-based collaboration scenarios [8].

Since Schelling’s seminal work in 1960 [4] many experiments have shown that people somehow manage to converge on a solution more effectively than what was predicted by the game-theoretical analysis. Apparently, for different reasons, some equilibria solutions appear more prominent than others for the players in the game. These solutions are denoted as focal points. In contrast, the game-theoretical framework fails to explain people’s decisions in such games [1], mostly because the problem of deciding between multiple Nash equilibria, which is one of the major challenges of game theory [4, 9].^1^ However, as Schelling showed in his experiments players do manage to converge on a same solution by relying on salient labels that help distinguish a set of more prominent solutions, which are the focal points [10, 11]. Thus, game theory lacks the ability to capture cognitive heuristics that people apply when solving coordination problem, such as symmetry, proximity, or extremeness [12, 13]. Therefore, experiments in behavioral game theory try to fill this gap by constructing cognitive models describing decision-making heuristics (e.g., [14–18]).

Another question that has been left unanswered is the degree of heterogeneity in peoples’ ability to successfully coordinate in tacit coordination games. In other words, given a set of tacit coordination problems, it seems that some people manage to successfully coordinate most of their answers with the unknown partner, while others experience difficulties in doing so. This ability to succeed in tacit coordination tasks was hinted at by Bacharach in [1] and was named “Schelling’s competence”. In other words, people who manage to coordinate most of their answers are regarded as having high Schelling’s competence. The variation in the level of Schelling’s competence might be explained by the propensity of applying multiple salient selection rules. The weighted combination of the selection rules was denoted as a strategic profile [15, 16, 19]. Each strategic profile reflects the subjective preferences of individual players regarding a set of heuristics or prominent and salient selection rules [15, 16, 19]. Thus, a strategic profile may be regarded as a weighted combination of the different selection rules utilized by a specific player across different game instances to achieve a successful coordination.

The overarching aim of our study was to model the relationship between individual coordination ability and decision-making within the framework of a tacit coordination game. To that end, we have conducted a large-scale tacit coordination experiment to collect behavioral data. We have first described the variability in individual coordination ability (iCA) [16, 17] among the players, detected the predominant selection rules (e.g., spatial proximity) that players have utilized, and finally modeled the relationship between iCA and the individual strategic profiles [16].

Next, we have validated the proposed model by predicting the individual coordination ability of a player based on their constructed strategic profile. Understanding the differences in individual’s tacit coordination abilities as well as their unique strategic profiles will allow a better prediction of human behavior in tacit coordination scenarios and consequently the improvement of algorithms for human–machine interactions.

## Methods

### Experimental design

**Fig. 1:**
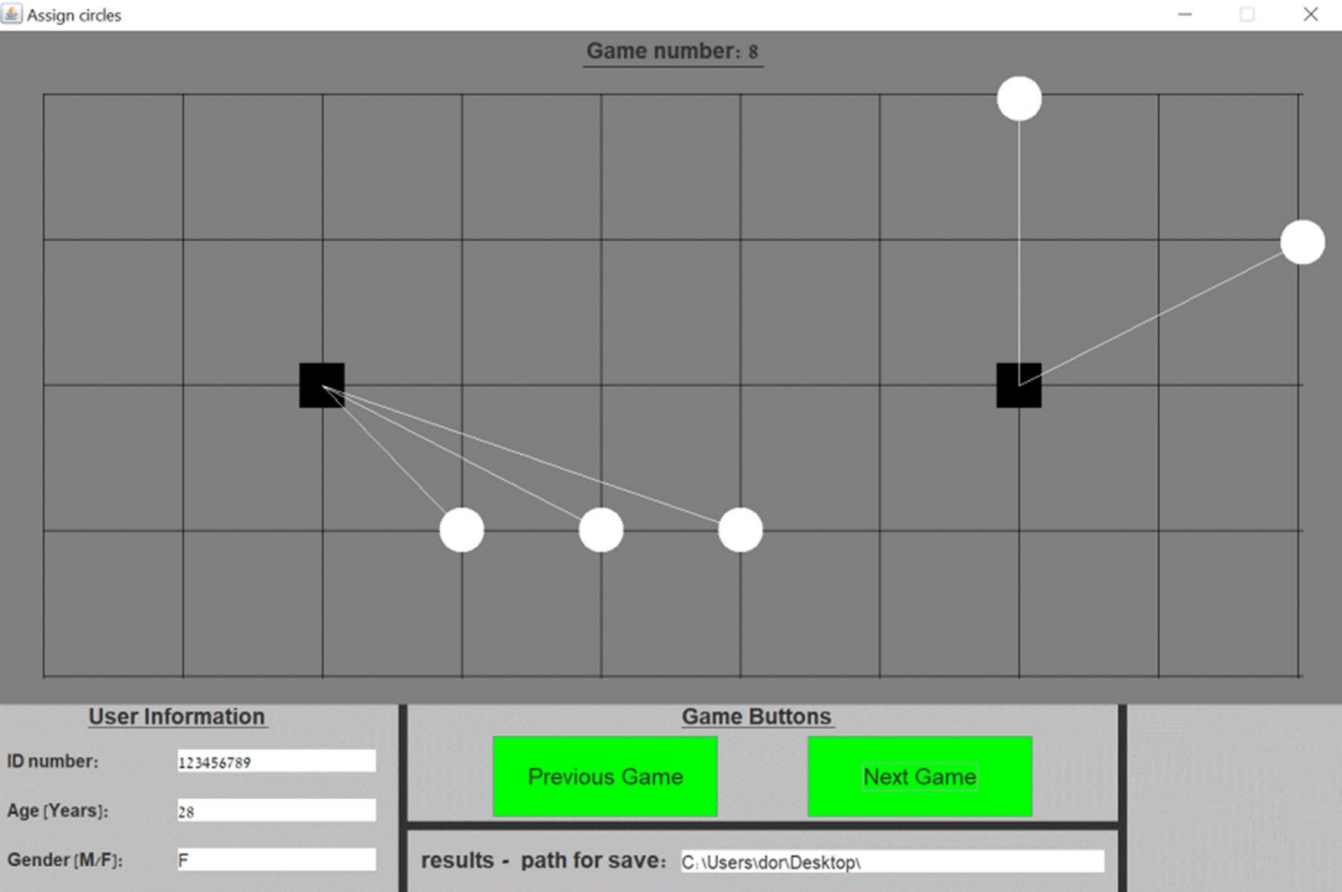
“Assign Circles” application window

To test the coordination ability of players we used the “Assign Circles” [12, 13, 15, 20] tacit coordination game. The players were presented with 14 different “Assign Circles” decision problems, each associated with a different board layout: 10 predefined problems were taken from [12] and additional 4 were randomly generated layouts. To avoid consistent bias that may have been caused by the order of the games, the games were presented randomly for each of the players. In each of the decision problems the players were asked to assign circles to squares with the aim of coordinating their assignment with an unknown player, who was presented with the same board layout (see Fig. 1 for a game example). That is, *a successful coordination was achieved when both players attached all the circles to the same squares*. In case of a successful coordination both players gained a point and each player could accumulate additional points as the game progressed. Both players had no communication capability at all, and their results were only revealed after all the games had been completed.

Based on [12, 13, 15, 16] it was assumed that participants would utilize three main selection rules when playing the “Assign Circles” games, as follows: (1) *closeness*—assigning each circle to the closest available square; (2) *accession*—assigning circles which are close to one another to the same square; and (3) *equality*—assigning the same number of circles to each of the two available squares. We examined the variability in how these three rules were weighted and aggregated as part of the decision-making process of each individual participant, for the “Assign Circles” game. Importantly, we do not claim that these three, domain dependent rules, represent an exhaustive listing of all the rules that are available for this game (see [12, 16, 18, 21–23]). Rather, we merely suggest that these three prominent selection rules should provide enough variability in the individual strategic profiles.

As the first ten decision problems of the game have a fixed layout, we can analyze each one of them using the above mentioned selection rules and detect the expected solution by implementing each of the rules in each game. In this way, we may potentially predict the solutions by which the players will choose to establish a focal point had we known the strategic profile of each participant and assuming consistency between games. For example, let us examine the game presented in Fig. 1. First, it is evident that the *equality* rule is not applicable as there are 5 circles and any division of either 2–3 or 3–2 will not enable the utilization of this rule. Second, the *accession* rule solves the coordination problem as illustrated in Fig. 1, which displays two distinct groups of circles. Lastly, using the *closeness* rule we expect the middle circle to be connected to the right-hand square rather than to the left one as displayed in Fig. 1. In a similar manner we can describe the resultant solution attained by using each of the selection rules for each game instance.

### Participants

The participants were 93 students that were enrolled in one of the courses on campus [49 of whom were female, mean age  = 22.93 (years), SD  = 1.97]. The 93 participants were randomly assigned to two consecutive sessions, with 48, and 45 participants in each of the sessions, respectively. All students were seated in one class and each student sat in front of a desktop monitor. Before the onset of the experiment, participants received an explanation regarding the overarching aim of the study, the experimental procedure, and the graphics of the application window. As participants were rewarded according to their performance on the “Assign Circles” task, they have also received an explanation regarding the criteria for allocation of rewards (in the form of course credit) according to how many points they gained. To verify that participants understood the concept of coordination, they were given several training examples by presenting screenshots of different layouts of discs for each of the players. Moreover, prior to the start of the session, several questions were asked by the experimenter regarding the usage of the application to ensure that participants know how to operate it.

The study was approved by the IRB committee of Ariel University. All participants provided verbal informed consent for the experiment.

### Measures

#### Coordination index (CI)

The CI measure proposed by Mehta et al. [12] is a statistical measure that allows determining the difficulty of coordination in a specific game. Specifically, the higher the CI, the easier it is to coordinate between two random players. Consider a coordination game with the set of possible solutions $L=\left\{{l}_{1},\dots ,{l}_{n}\right\}$, where a solution is an implementation of specific strategies, and $N$ is the number players, when each of them plays the game only once with an unknown anonymous partner. For each solution ${l}_{j}$ let ${m}_{j}$ be the number of individuals who choose it, then the coordination index c is given by:

1 $$ c = { }\frac{{\mathop \sum \nolimits_{j} m_{j} \left( {m_{j} - 1} \right)}}{{N\left( {N - 1} \right)}}. $$

This index measures the probability that two distinct individuals, chosen at random without replacement from the set of N individuals, choose the same solution. It takes the value 1 if all individuals selected the same single solution and 0 if everyone selected a different solution. If solutions are chosen at random, the expected value of the index is $\frac{1}{n}$.

#### Individual coordination ability (iCA)

To measure the coordination ability of each player, we assessed their ability to coordinate with all 92 other participants in the experiment rather than with a single random participant. For this purpose, we calculated the total number of games in which each player was able to coordinate their responses against the entire experimental population and normalized it by the total number of games. It should be noted that the calculation was only carried out on the ten predefined games, but not on the four randomized games, since only the predefined games were kept constant across participants. iCA [16, 17, 24] is formally defined as follows:

2 $$ {\text{iCA}}\left( i \right) = \mathop \sum \limits_{{j = 1{ }|{ }\left( {j \ne i} \right)}}^{N} \mathop \sum \limits_{k = 1}^{t} \frac{{{\text{CF}}\left( {i,j,k} \right)}}{{\left( {N - 1} \right){*}t}}, $$

where *i* denotes the *i*th participant, *j* denotes the index of the *j*th co-player, *N* denotes the total number of participants, and *t* denotes the number of games in the experiments. The CF (coordination function) is defined as follows:

3 $$ {\text{CF}}\left( {i,{ }j,{ }k} \right) = \left\{ \begin{gathered} 1; \;{\text{if players}}\;i{\text{ and }}j{\text{ chose the same label in game }}k \hfill \\ 0;\;{\text{otherwise}}{.} \hfill \\ \end{gathered} \right. $$

The iCA measure is not intended to be an absolute score, but rather it allows ranking the participants that completed the same set of tasks based on their iCA values as was the case in our study.

#### Strategy rate (SR)

The strategy profile is described by the weighted combination of the selection rules. To measure the frequency of choosing each of the abovementioned strategies by a single player, we first defined the strategy rate (SR). SR is defined as the probability that a specific player will choose using one of the three selection rules in one of the decision problems included in the Assign Circles game. The SR was calculated based on the behavioral performance data of each player in each decision problem. To compute the SR measure, we first defined a Game Tag (GT) variable for each strategy, which can have one of three different values as follows:

4 $$ {\text{GT}}(i,\;k)_{{{\text{strategy}}}} = \left\{ \begin{gathered} {\mathbf{1}},\;{\text{if the strategy was available in the }}k{\text{th}}\;{\text{game and the }}i{\text{th player used it}} \hfill \\ {\mathbf{0}},\;{\text{if the strategy was not available in the }}k{\text{th game}} \hfill \\ {\mathbf{ - 1}},\;{\text{if the strategy was available in the }}k{\text{th}}\;{\text{game and the }}i{\text{th player did not use it}} \hfill \\ \end{gathered} \right.. $$

The SR for each of the three game strategies can then be calculated as follows:

5 $$ {\text{SR}}\left( i \right)_{{\left\{ {{\text{Acc}},\;{\text{Equ}},\;{\text{Clo}}} \right\}}} = \frac{{\mathop \sum \nolimits_{k = 1}^{14} \left[ {{\text{GT}}\left( {i,\;k} \right)_{{\left\{ {{\text{Acc}},\;{\text{Equ}},\;{\text{Clo}}} \right\}}} = 1} \right]}}{{\mathop \sum \nolimits_{k = 1}^{14} \left| {{\text{GT}}\left( {i,\;k} \right)_{{\left\{ {{\text{Acc}},\;{\text{Equ}},\;{\text{Clo}}} \right\}}} } \right|}}. $$

Together, the strategic profile of each individual player is a vector composed of three elements, one for each of the selection rules [(Acc, Equ, Clo)] using Eq. 5. For player #7, for example, the number of games in which each of the strategies (Closeness, Accession, and Equality) was applicable was 8, 10, 7, respectively, and the number of games in which each of the strategies was implemented was 5, 4, and 4, respectively. Hence, the corresponding obtained SR values were as follows: 5/8 = 0.63; 4/10 = 0.40; 4/7 = 0.57.

## Results

Before examination of the results pertaining to the iCA scores and the strategic profiles, it is important to demonstrate that the players were motivated to coordinate with the other unknown partner, that is, that a solution in each of the games was not randomly selected. Table 1 compares the observed CI scores with the hypothetical CI scores that would have been obtained had all the players randomly picked their solution, as expected by game theory. We can see that all decision problems were associated with a significantly higher CI values than random picking (a higher score denotes a better ability to coordinate). We can also notice that the difficulty of coordination varies across the different decision problems, as reflected by the fluctuations in the CI score. There are few easy coordination problems (e.g., #1, #2), few hard problems (e.g., #3, #8) and few problems with a medium difficulty level (e.g., #4, #5). Recall that the ICA is a relative measure and is calculated by pairing each player with each of the other players in each coordination problem. Therefore, all players have encountered the exact same set of predefined problems (1–10).

**Table 1:** Comparison of the observed CI scores to random picking and selection rules utilization ratios

Game number	1	2	3	4	5	6	7	8	9	10
CI	0.71	0.75	0.36	0.43	0.55	0.52	0.74	0.36	0.33	0.41
Random picking CI	0.25 (1/4)	0.0625 (1/16)	0.125 (1/8)	0.0625 (1/16)	0.125 (1/8)	0.0625 (1/16)	0.5 (1/2)	0.25 (1/4)	0.0625 (1/16)	0.0313 (1/32)
Closeness utilization	0.8387	0.8709	Not applicable	Not applicable	0.7419	Not applicable	Not applicable	Not applicable	0.3333	0.6129
Equality utilization	0.8387	0.8709	Not applicable	0.6451	Not applicable	0.7204	Not applicable	Not applicable	0.4731	Not applicable
Accession utilization	0.8387	0.8709	0.3225	Not applicable	0.7419	0.0860	Not applicable	Not applicable	0.3333	0.2043

**Fig. 2:**
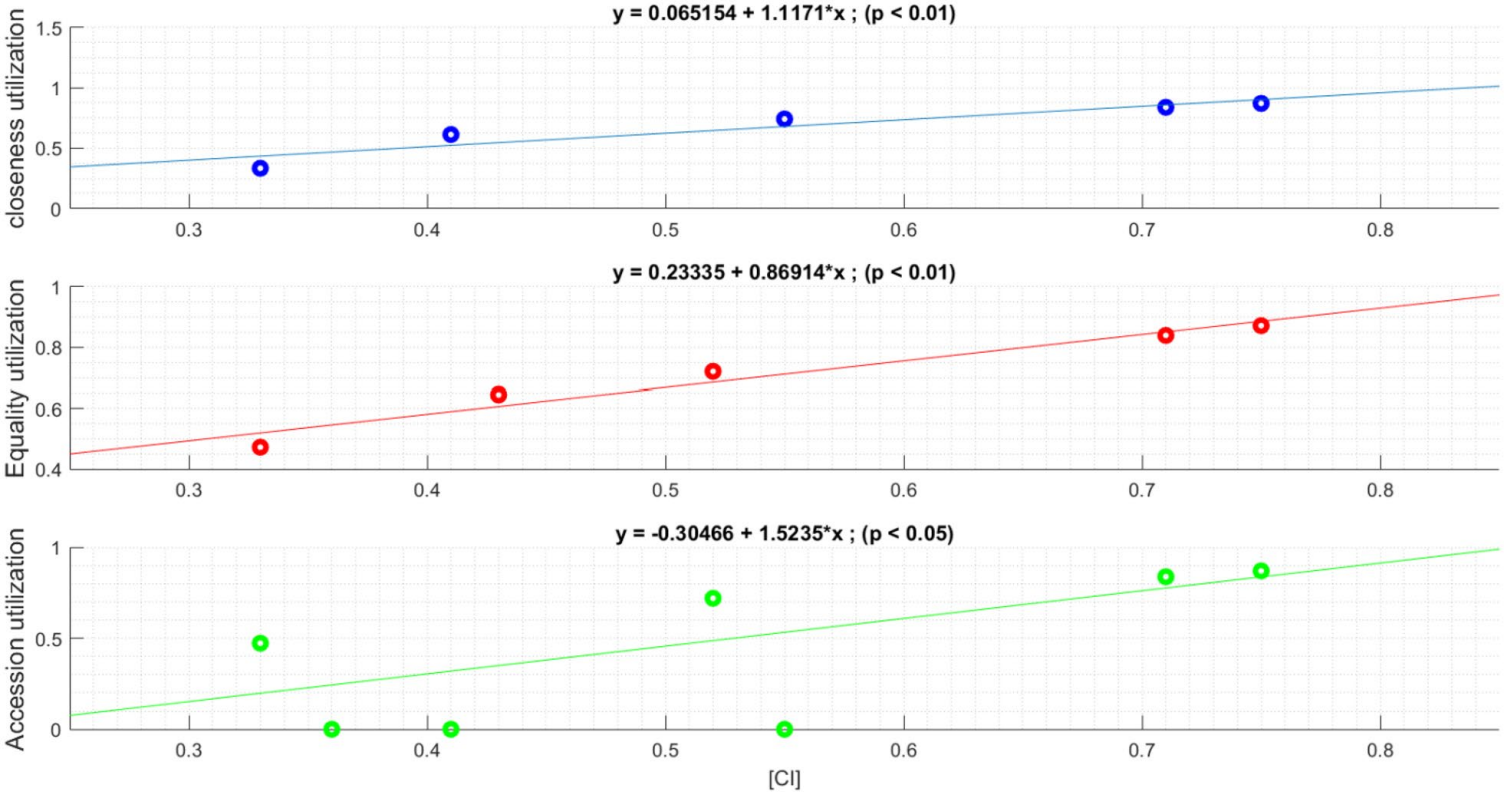
The relationship between CI and the frequency of implementation of the selection rules

Figure 2 shows a regression analysis of the data shown in Table 1. Figure 2 shows that there is a positive relationship (*p*  < 0.05) between the three main selection rules and the level of difficulty of the game, which is measured by the CI value. The higher the CI the easier is the game and the percentage of selection rule implementation.

### Characterizing the variability in iCA scores

**Fig. 3:**
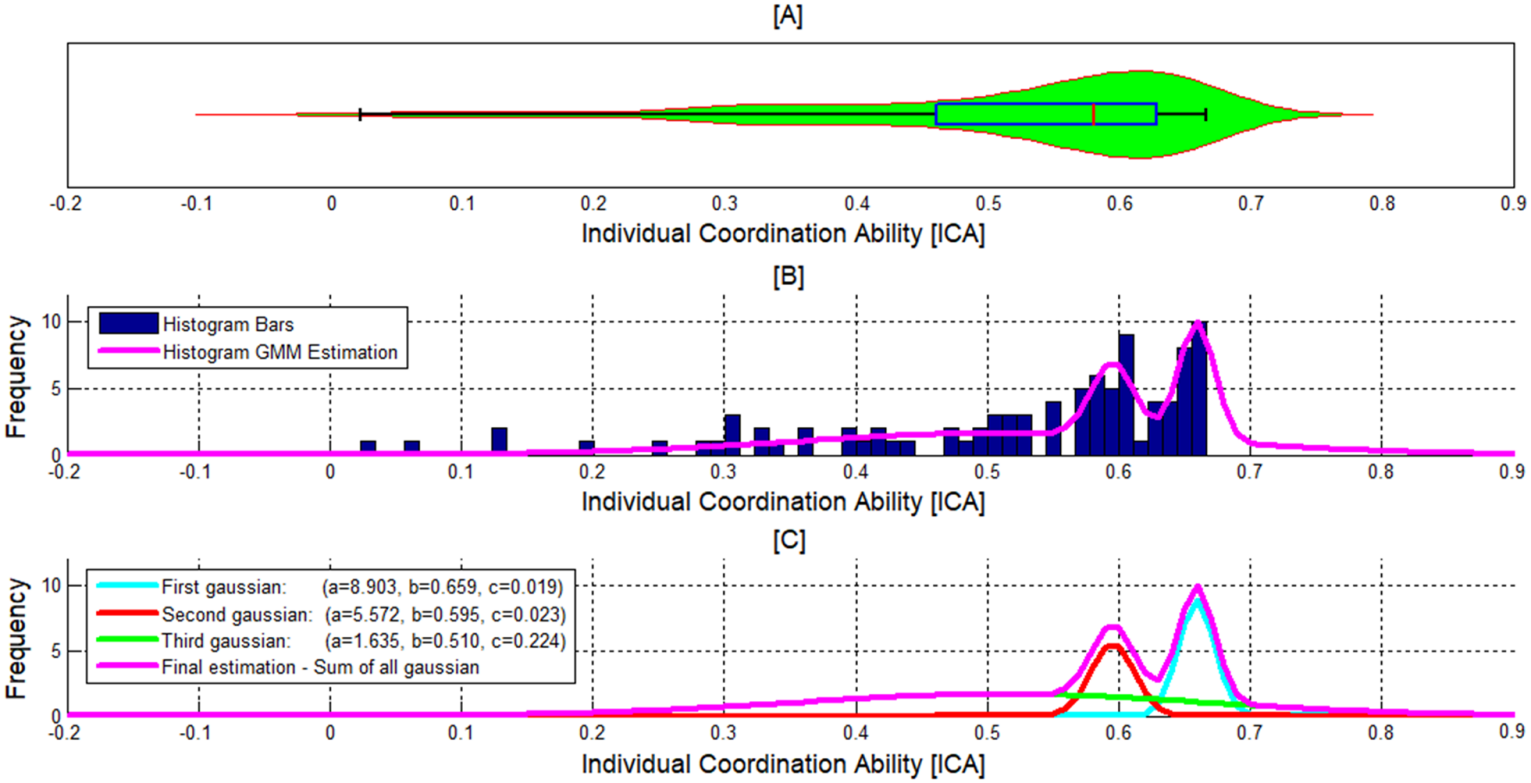
The distribution of the iCA scores. **A** Violin plot with boxplot. **B** Histogram. **C** Gaussian mixture model estimation. The abscissa is the ICA score. Note that the third Gaussian (green line) is obscured by the general model because of its high variability

To characterize the variability in the coordination ability among different players, we have plotted the distribution of the data using a violin plot combined with boxplot, a histogram and a Gaussian mixture model (GMM) (Fig. 3A–C, respectively). It can be observed that the range of the iCA score is very diverse: the lowest score was 0.023 (average of 2% successful coordination) and the highest score 0.666 (average of 66% successful coordination). Also, we can see that the range of the iCA score was smaller in the case of the top players (0.55, 0.7) and therefore this group was more homogeneous compared to the lower quartile. It is also evident that the distribution is negatively skewed and that the lower 25% iCA scores are more spread out than the higher scores (Fig. 3A). In addition, the Dip test of unimodality [25] indicated that the distribution is multimodal (*p*  > 0.1, Dip  = 0.025) while by estimating the distribution by a GMM with K Gaussians it was demonstrated that the entire distribution can be described by three Gaussians (*k*  = 3 was determined by using Residual sum of squares). The mathematical formulation of the iCA mixture model, which is presented graphically in Fig. 3C, can be described using the following formula:

6 $$ {\text{iCA}}\left( x \right) = 8.903{\text{*e}}^{{ - \left( {\frac{{\left( {x - { }0.659} \right)}}{0.019}} \right)^{2} }} + 5.572{\text{*e}}^{{ - \left( {\frac{{\left( {x - { }0.595} \right)}}{0.023}} \right)^{2} }} + 1.635{\text{*e}}^{{ - \left( {\frac{{\left( {x - { }0.510} \right)}}{0.224}} \right)^{2} }} . $$

It is noteworthy that this division into *k*  = 3 was also corroborated using the silhouette index [26] for *K* values at the range [1, 10]. The three Gaussians composing the distribution (Fig. 3C) comprise the third Gaussian with the lowest iCA scores, which corresponds with the lower 25%, the second Gaussian which surrounds the median, and the first Gaussian which corresponds with the upper portion of the IQR as well as with the upper quartile (Fig. 3A, B).

### Detection of dominant SR values in the strategic profile

Before analyzing the relationship between iCA and the strategic profile, it is first necessary to identify the predominant selection rules utilized by each of the players. Table 2 displays the distribution parameters of each of the three SR indices:

**Table 2:** Strategy rate statistics

	SR_Closeness_	SR_Equality_	SR_Accession_
Mean	0.6919	0.6685	0.3439
Standard deviation	0.2690	0.2624	0.1569
Median	0.7500	0.7143	0.3636

It is clear from Table 2 that there were two leading selection rules: closeness, and equality. The median probability of using each of the two selection rules by a random player was over 70%, if they were applicable in the game. In contrast, the accession rule had a much lower probability of selection on average by a random player.

A one-way analysis of variance showed that the main effect of strategy rate was significant [*F*(2, 90)  =  63.67, *p*  < 0.001]. Post hoc testing (Tukey HSD) [27] indicated that the average mean value of the accession SR value was significantly lower than each of the other two SR values, closeness, and equality (*p*  < 0.001).

### Modeling the relationship between strategic profile and iCA

The previous section showed that the strategic profile contains two leading strategies (closeness and equality) and another secondary strategy (accession). This phenomenon may affect the performance of the predictive model due to unwanted dependencies between the independent variables (SR_Closeness_, SR_Equality_, SR_Accession_). On the other hand, the omission of one of these variables results in discarded data that may consequently lead to impaired performance of the predictive model. To deal with this problem we will use a dimensionality reduction technique [28–30] to maximize the variance of the data in a smaller number of features, thereby reducing the dependencies among the variables. To do so we used the principal component analysis (PCA) algorithm [29]. We have first conducted feature selection and then performed feature extraction, i.e., we have constructed a new feature space by data compression [29, 31].

**Fig. 4:**
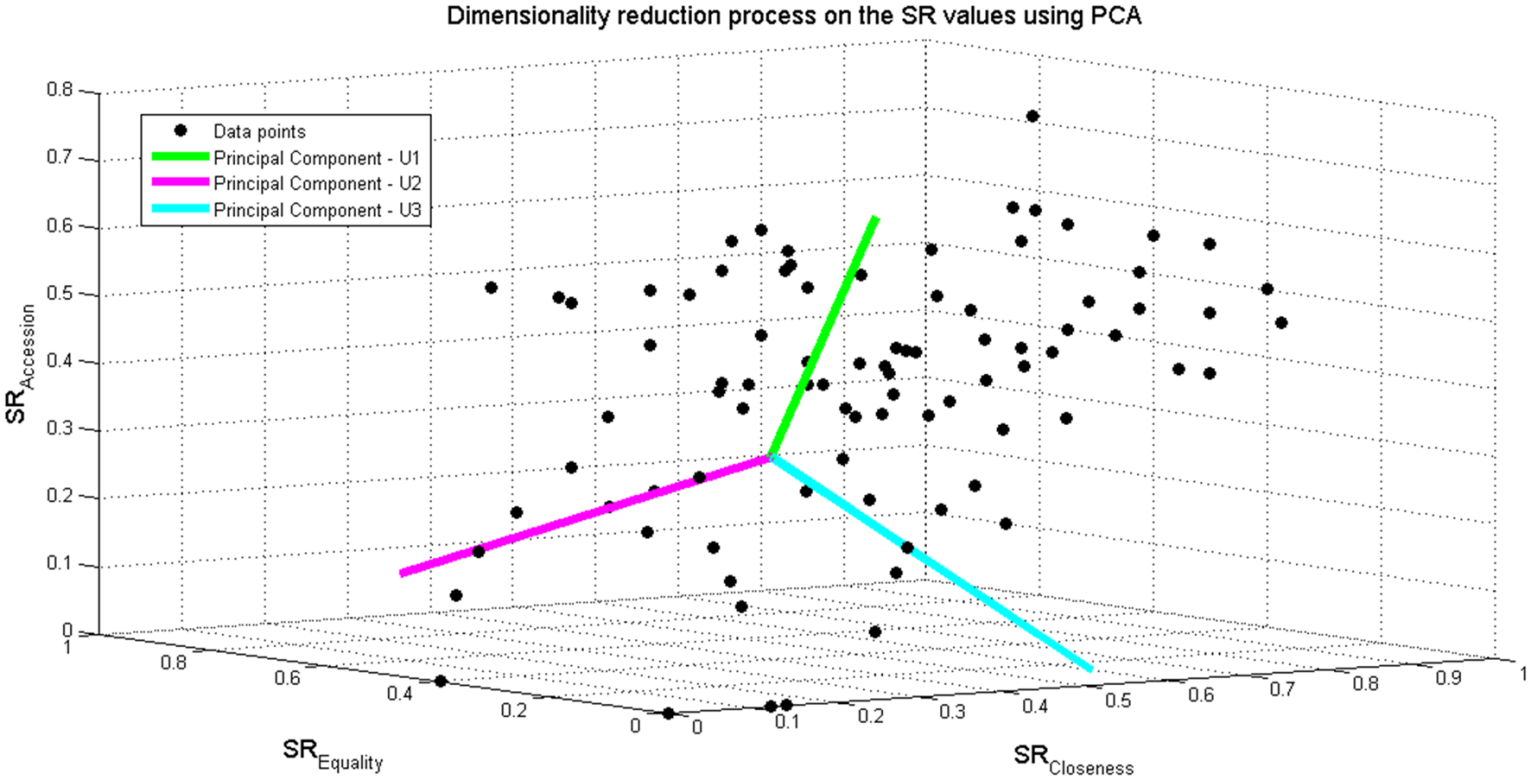
Applying dimensionality reduction to the SR values by PCA. All three SR points of each player (closeness, equality, and accession) are displayed within a 3D plane, together with the principal components obtained following dimension reduction

For feature selection we have first extracted the new base vectors, namely, the principal components. The components were extracted by calculating the eigenvectors of the covariance matrix of the data after conducting mean and variance normalization. Then, the principal components were sorted according to the magnitude of their coefficients (i.e., eigenvectors) from largest to smallest (in absolute values). Figure 4 presents the three principal components (each denoted by *U*) overlaid on the original data (based on the three SR values). Next, we performed feature extraction and selected the two components, *U*1 and *U*2 with the highest explained variance (i.e., with the two largest eigenvalues) to create a new dimensional feature space. Consequently, we have projected the original data onto a new feature space while retaining the maximum variance in the original dataset (Table 3). Thus, we have compressed the original 3D data into a new 2D space. Specifically, two new data features have been produced: $S_{1}$—the projection of the vector *x* on *U*1, and $S_{2}$—the projection of the vector *x* on *U*2.

**Table 3:** Retained variance in dimension reduction process

Number of principal components	1	2	3
Retained variance (%)	70.74	90.43	100

To ensure that no critical data were lost during the process of dimensionality reduction, we performed a reconstruction of the reduced data into the original dimension while comparing the reconstructed data to the original data vector before compression. The strategic profile of the *i*th player is denoted by *x*^(*i*)^ vector, which contains the three SR values. The retained variance calculation was performed as follows:

7 $$ {\text{Retained variance}} = 1 - { }\frac{{\frac{1}{m}\mathop \sum \nolimits_{i = 1}^{m} \left| {\left| {x^{\left( i \right)} - x_{approx}^{\left( i \right)} } \right|} \right|^{2} }}{{\frac{1}{m}\mathop \sum \nolimits_{i = 1}^{m} \left| {\left| {x^{\left( i \right)} } \right|} \right|^{2} }}. $$

The calculation for each number of selected principal components produced the following results, described in Table 3.

As can be seen in Table 3, for two principal components new data vectors can be produced that contain about 90% of the variance of the original data, where the correlation between the two variables is negligible (due to the dimensionality reduction process).

Following data compression, we performed a regression with the single dependent variable being the coordination ability of the *i*th player, described by the iCA score, while the predictors were the newly found *S*1 and *S*2 features. In order to find the model coefficients, we used multiple linear regressions:

8.1 $$ {\text{ICA}}\left( i \right) = 0.52118 + 0.090788{*}S_{1} + 0.054041{*}S_{2} , $$

8.2 $$ R^{2} = 0.8733{ };F = { }310.0768{ };{ }p < 0.001{ };{\text{ VAR}}_{{{\text{error}}}} = 0.0029, $$

where

8.3 $$ S1 = { }0.610*{\text{SR}}_{{{\text{Closeness}}}} + { }0.518*{\text{SR}}_{{\text{Equality }}} + { }0.599*{\text{SR}}_{{{\text{Accession}}}} , $$

8.4 $$ S2 = { } - 0.312*{\text{SR}}_{{{\text{Closeness}}}} + { }0.853*{\text{SR}}_{{\text{Equality }}} + { } - 0.418*{\text{SR}}_{{{\text{Accession}}}} . $$

**Fig. 5:**
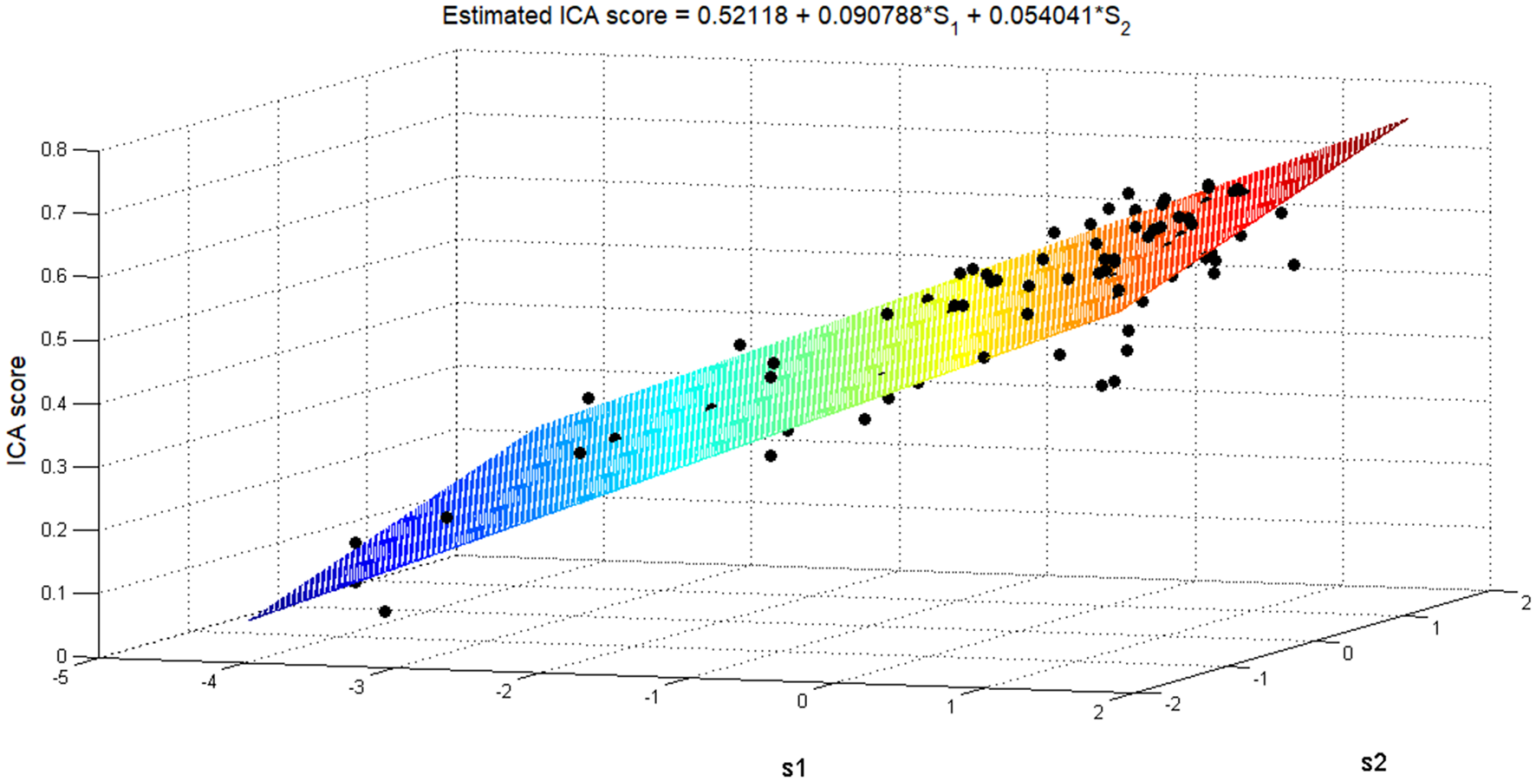
The regression model in a 3D space. A multi-variable regression model that describes the relationship between the three SR variables and iCA. Each dot represents the iCA value of each player as a function of their strategic profile after data compression (3D to 2D). The new model is presented as a 2D plane in a 3D space

We can see from Eq. 8.2 the strong correlation between iCA and the strategic profile, while our correlation is based only on two variables, $S_{1}$ and $S_{2}$. This allows us to draw a 2D plane, which represents the model, in a three-dimensional space overlaid on the corresponding iCA values, as can be seen in Fig. 5.

### Model validation

In the previous section, we presented a model (Eqs. 8.1, 8.2, 8.3, 8.4) that links between the iCA scores and the individual strategic profiles of the players. In this section, we present validation results to assure that the model can be applied on a new dataset that was not used for model construction. Here, we demonstrate the robustness of the model by predicting the iCA values of players based on their individual strategic profile.

#### Participants

The participants in the validation group were 33 students that were enrolled in one of the courses on campus [12 of whom were female, mean age  = 24.23 (years), SD  = 2.21]. The validation group participated in the same experimental design as the original group (see Sects. 2.1 and 2.2).

#### iCA distribution

**Fig. 6:**
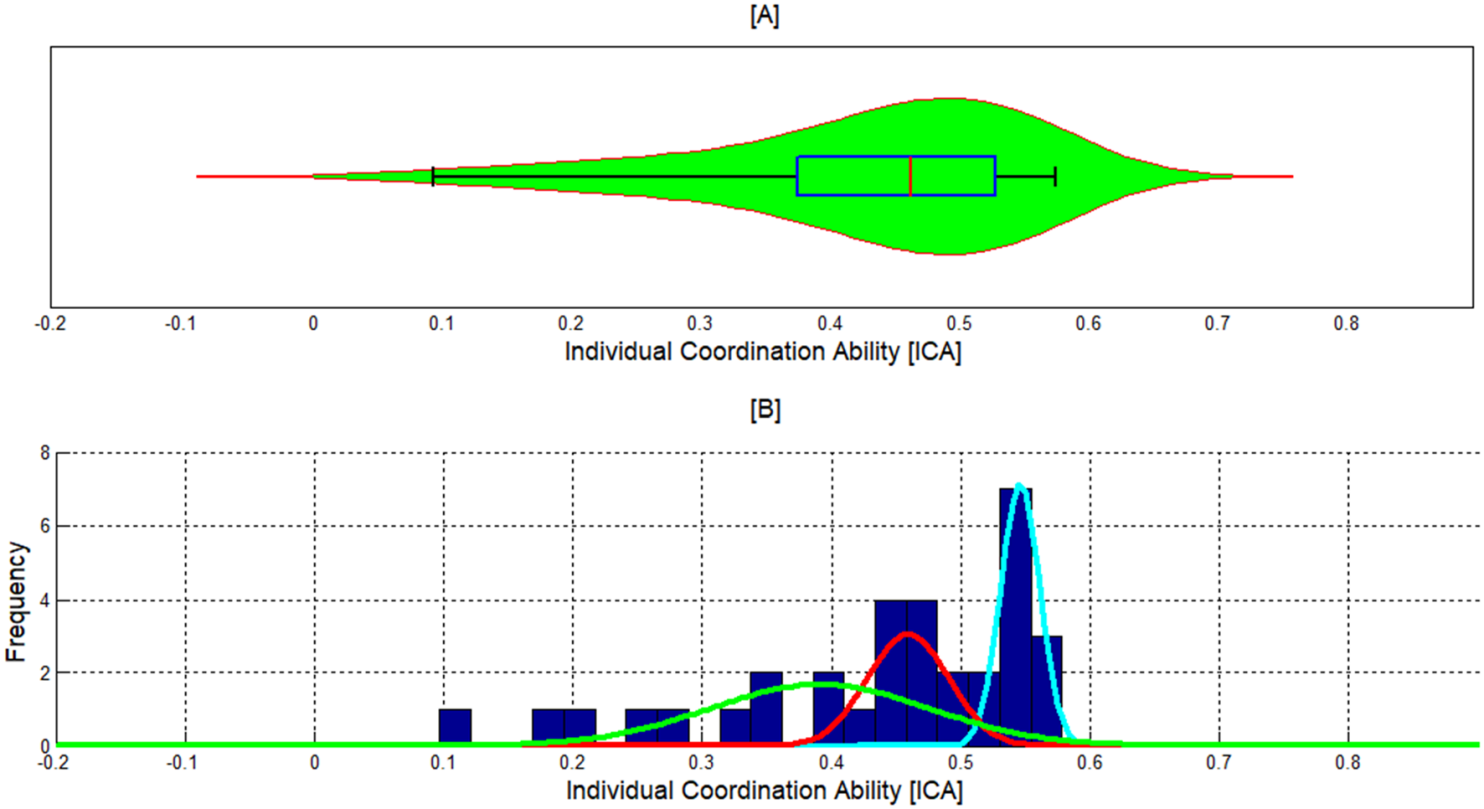
ICA score distribution of the validation group. **A** Violin plot with boxplot. **B** Histogram with Gaussian mixture model estimation

Figure 6 displays the iCA score distribution of the validation group. It can be seen that the iCA distribution of the two groups (original group and validation group) is very similar (compare Fig. 6 with Fig. 3). To validate this apparent similarity, the iCA distribution of the validation group was estimated by a GMM. It was demonstrated that the validation group was characterized with the same iCA distribution as the original study group. That is, the entire distribution of iCA scores could also be described by three Gaussians (*k*  = 3) and a similar partitioning into sub-groups according to the level of coordination ability was observed (Fig. 6A, B). That is, there is a group of players with a high coordination ability (blue Gaussian, coinciding with the upper quartile), a group of players with an intermediate coordination ability (red Gaussian, surrounding the median) and a group of players with a low coordination ability that manifests a wide dispersion (green Gaussian, coinciding with the lower portion of the IQR and the lower 25%).

#### Prediction of the iCA score and model evaluation

To assess the accuracy of the proposed prediction model (Eq. 8.1) we have examined the statistical indices of the relative error between the predicted value estimated by the model and the real value calculated using the iCA formula (Eq. 2). The relative absolute error is defined as follows:

9 $$ {\text{iCA relative}}\;{\text{absolute}}\;{\text{error}} (\% ) = \frac{{\left| {{\text{iCA}}_{{{\text{real}}}} - {\text{iCA}}_{{{\text{predicted}}}} } \right|}}{{{\text{iCA}}_{{{\text{real}}}} }}*100. $$

Table 4 presents the percentiles of the distribution of the ICA relative absolute error. It can be observed that the median relative absolute error was only 7.811% while 75% of the total population does not exceed an error of 13.314%. In addition, out of 33 predictions, there were only 3 values for which the relative absolute error was greater than 30%.

**Table 4:** Percentiles of the iCA relative absolute error distribution

25th percentile	50th percentile (median)	75th percentile
3.915	7.811	13.314

These results demonstrate that the predictive model is robust and can be generalized to an independent dataset. That is, the estimated iCA values of a player can be predicted based on their strategic profile with high accuracy.

## Discussion

This research contributes to key issues pertaining to the process of decision-making in tacit coordination. First, although in previous studies the variability among players has been examined in different domains (e.g., [18, 32]), in our study the variability was explored at the individual level of analysis in the domain of tacit coordination games. Second, to find the predominant selection rules that were applied during task performance, we devised the SR index that was computed for each individual player. This analysis hinted to the existence of two predominant selection rules: closeness and equality. This indicates that certain selection rules are preferred by players and are more useful for coordination than others in the context of a specific task. Third, we devised a novel method for constructing a strategic profile for each individual player. The method relies on the projection of the individual SR indices onto a 3D strategy space. Finally, we have used multiple regression analysis and showed that there is a strong correlation between the player’s strategic profile and their coordination ability.

Previous research dealing with focal points examined a wide range of selection rules in different games (e.g., [12, 13, 18, 33]). In these previous studies, the data were processed and analyzed at the group (aggregate) level only, and so no relationship was demonstrated between individual coordination ability and the set of strategies implemented during the game as was done here. Yet in several other studies [15, 16, 20] an individual level of analysis has been implemented. However, in these studies the emphasis was on examining the effect of social factors (e.g., culture [15], social value orientation [20, 34] and loss aversion [35]) on strategic behavior whereas the current study is more generic as it does not utilize any prior knowledge about the players.

Importantly, while our work shows the ability to model individual coordination ability and use it to predict behavior, our framework is demonstrated by using only one domain and its generalizability is yet to be explored. For example, given a new coordination domain, the selection rules have to be defined first and only then our model could be applied. The preprocessing step of finding the selection rules in a given domain is still lacking in the absence of a formal procedure for extracting the selection rules.

Thus, the main contribution of this study is the construction of a model predicting individual coordination ability although this dependent variable was characterized by a multimodal distribution (see Figs. 3, 5). This multimodal distribution might corroborate cognitive hierarchy theory [36–38], which postulates that individuals differ in their depth of reasoning. By this account, an agent is bounded by the *k* steps of reasoning they can perform [39, 40]. Thus, each of the three Gaussians found in the current study may correspond to a different level k that bounds the best response given by the players. Hence, the second and third Gaussians found in our study may each correspond to a level *k*  ≥ 1, while the first Gaussian probably includes a substantial number of players with a level *k*  = 0 (see Figs. 3C, 6B). Noteworthily, the suggested connection between our findings and level *k* theory should be further explored especially in view of findings indicating inconsistencies in the level *k* of players, even in the same game, which imply that the depth of reasoning is stochastic in nature [41, 42].

Nevertheless, our results can also be compatible with a team reasoning approach [18, 43–46]. However, it was previously shown that coordination rate on a focal point depends on whether the game is symmetric or not as well as on stake size [47]. Therefore, the mode of reasoning of the player may be explained by either cognitive hierarchy or team reasoning, depending on specific game features [47].

The focal points that were modeled in our study are based on spatial properties (e.g., closeness, equality, and accession). Consequently, we expect the model we have constructed to be applicable to other contexts where focal points are based on spatial cues, e.g., “Bargaining Table” [14, 35, 40, 48] and “Moving Discs” [18]. However, in other cases, where focal points are based on non-spatial features (e.g., the “word Selection” task based on semantic meaning [18, 24, 42, 49]) our model should be modified accordingly. For example, by updating the number of Gaussians which correspond to different strategic profiles extracted from the game.

## Conclusions

In summary, in this study we investigated the distribution of individual coordination ability in tacit coordination games and constructed a predictive model based on the individual strategic profiles of the players. The strong relationship found in our study between the strategic profile and coordination ability suggests that players differ in the strength by which they prefer salient choices [50] and this in turn might affect their coordination ability. Our findings could be explained by either cognitive hierarchy or team reasoning. Nevertheless, they indicate that individual-level analysis is essential for gaining insight into the diversity among decision-makers regarding their strategic performance.

The results of the current study open several avenues for future research. First, it will be interesting to consider tacit coordination games of divergent interests (e.g., [33, 48]). That is, games in which the parties are required to coordinate their answers, while different outcomes of the game may yield different utilities for each of the players. Second, it is worthwhile examining the effect of personality traits and attitudes that correlate with coordination ability, such as introversion–extroversion [51], or social value orientation (e.g., [20, 52, 53]), on the strategic profiles of the players. Third, improved models in the framework of human–machine coordination [3, 54] can optimize performance in diverse contexts such as intension prediction [55] and safety [56] in industry, fraud detections (e.g., [57–59]), as well as in other complex tasks (e.g., [60]). Fourth, it might also be worthwhile exploring whether the differences in individual coordination ability are accompanied by parallel changes in brain activation reflected by electrophysiological markers associated, for example, with cognitive load [24] or with time pressure [50]. These electrophysiological markers could then be added to our model as additional features that may improve its predictive ability. Finally, it is possible that strategic profiles are better described by the complexity level of the game (i.e., number of possible solutions—2^#number of discs^) rather than by the selection rules themselves. Therefore, it is worthwhile to repeat the current study while the set of games is divided into distinct levels of coordination difficulty.

## Appendices

### Appendix A: “Assign Circles” predefined game boards

**Fig. 7:**
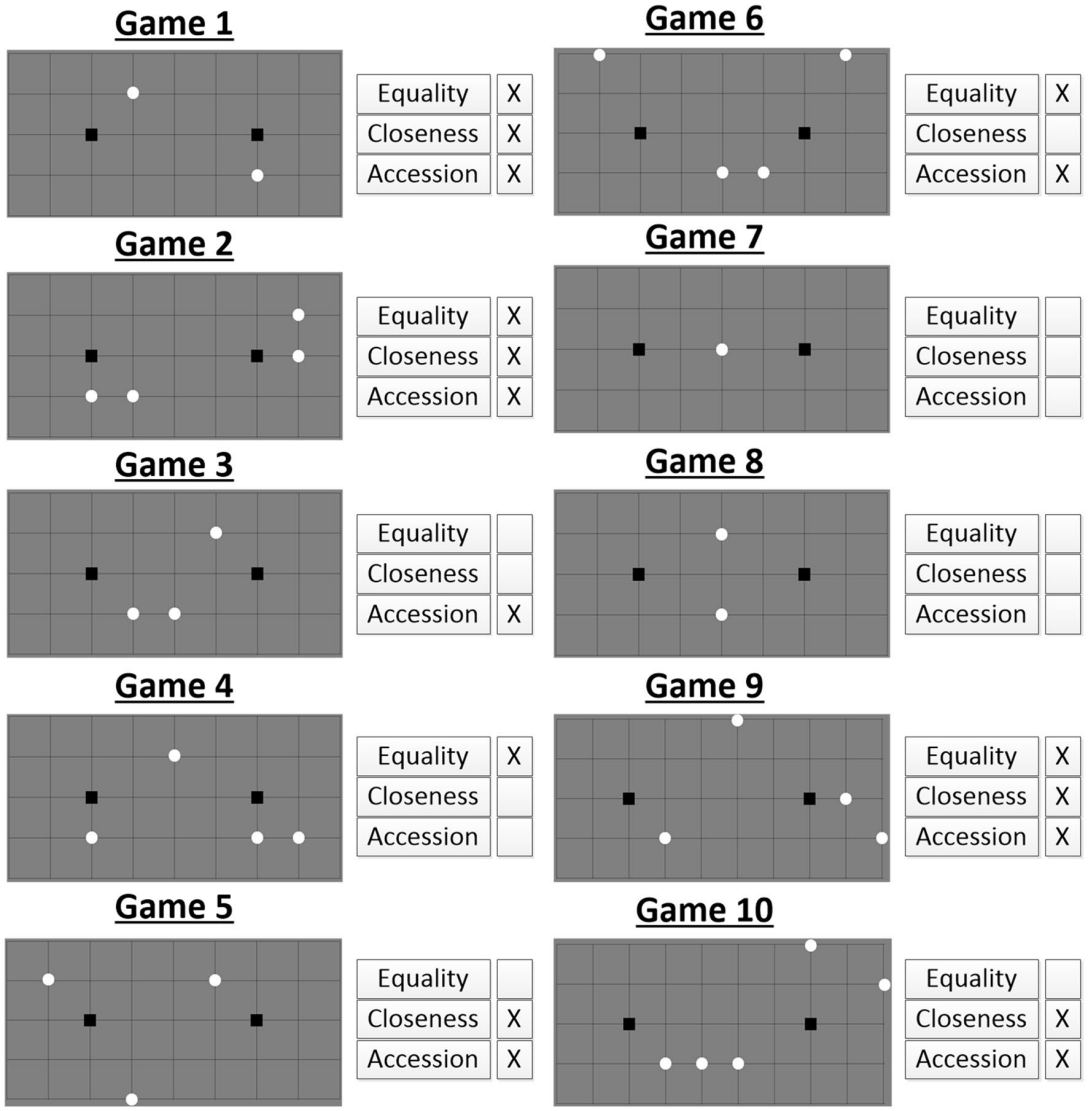
Diagrams presenting predefined games

The 10 predefined games are presented in Fig. 7. The board’s structure is based on the design of questions 11–20 in [12]. The order of appearance of the various games in the application was completely random. Next to each game board, the availability of the three main selection rules, which is used to construct the strategic profile, is presented.

### Appendix B: Selection rules implementation in the predefined games (#1–10)

In this section, we present the complete definition of the three selection rules. The following definitions are directly quoted from [12], pp. 173–175.

#### Closeness

For the rule of closeness to be applicable in a game, there has to be some commonly recognized concept of closeness of association between the two classes of objects (circles and squares). In the case of our grid, the most obvious and unambiguous measure of closeness of association between a circle and a square is the distance between them. Thus, we interpret the rule of closeness as: assign each circle to the nearer square.

#### Accession

The rule of accession implies that if a set of circles form a coherent group, all the circles in the group should be assigned to the same square. We shall say that two circles are connected if they are located at adjacent points in the grid, linked by a horizontal or vertical line, and we shall interpret a ‘coherent group’ as a set of connected circles. We define the distance between a square and a set of connected circles as the distance between the square and the nearest circles in the set. Then we interpret the rule of accessions the following formula: assign each set of connected circles to the nearer square.

#### Equality

The rule of equality suggests the general formula: if there is an even number of circles, assign half of them to the one square and half of them to the other square. As stated, this rule never implies a unique assignment of circles to squares; we posit the median line rule as a subrule of refinement of the rule of equality, which uses the metric of closeness to discriminate among equal assignments. This rule is: if there is a vertical line such that an equal number of circles lie on each side, then assign circles left of the line to the left-hand square, and circles right of the line to the right-hand square.

Table 5 in Appendix (taken from [12]) presents the specific solutions obtained by implementing each one of the three selection rules in games 1–10. Each one of the selection rules can only be implemented in a game board only if it defines a unique choice (there is only a single interpretation of the solution). In Table 5 “L” represents a connection of a circle to the left square and “R” to the right square. The circles are ordered from left to right and from top to bottom. For example, in game #3 the assignment implied by the rule of accession denoted by “LLR”, means that the two bottom circles are connected to the left square and that the upper circle is connected to the right square.

**Table 5:** Implication of the main selection rules

Game	Unique assignment implied by rule of			Predicted responses
	Closeness	Accession	Equality	
1	LR	LR	LR	LR
2	LLRR	LLRR	LLRR	LLRR
3	None	LLR	None	LLR
4	None	None	LLRR	LLRR
5	LLR	LLR	None	None
6	None	LRRR	LLRR	LRRR or LLRR
7	None	None	None	None
8	None	None	None	None
9	LRRR	LRRR	LLRR	LRRR or LLRR
10	LLRRR	LLLRR	None	LLRRR or LLLRR

## Data Availability

The datasets generated and/or analyzed during the current study, together with the corresponding analysis codes (which fits MATLAB R2016a) are available on the website of the “NeuroIS Lab” at Ariel University, (https://www.ariel.ac.il/wp/neurois/).

## Footnotes

1 Quoting Binmore ([61], p. 262), "*The equilibrium selection problem is perhaps the greatest challenge facing modern game theory*".
